# Real-time Analysis of Auxin Response, Cell Wall pH and Elongation in *Arabidopsis thaliana* Hypocotyls

**DOI:** 10.21769/BioProtoc.2685

**Published:** 2018-01-05

**Authors:** Lanxin Li, S. F. Gabriel Krens, Matyáš Fendrych, Jiří Friml

**Affiliations:** Institute of Science and Technology (IST) Austria, Klosterneuburg, Austria

**Keywords:** Auxin signaling, Cell wall pH, Cell elongation, Hypocotyl, Live-cell imaging

## Abstract

The rapid auxin-triggered growth of the *Arabidopsis* hypocotyls involves the nuclear TIR1/AFB-Aux/IAA signaling and is accompanied by acidification of the apoplast and cell walls (Fendrych *et al*., 2016). Here, we describe in detail the method for analysis of the elongation and the TIR1/AFB-Aux/IAA-dependent auxin response in hypocotyl segments as well as the determination of relative values of the cell wall pH.

## Background

Phytohormone auxin induces rapid growth in *Arabidopsis thaliana* hypocotyls. This process requires the TIR1/AFB-Aux/IAA auxin co-receptor. Auxin promotes the binding of TIR1/AFB and Aux/IAA, which leads to ubiquitination and degradation of the latter, and results in transcription of auxin-responsive genes. This protocol focuses on measuring the growth, auxin signaling and cell wall acidification in *Arabidopsis thaliana* etiolated hypocotyls. This protocol is based on the previous work of Schenck *et al.*, 2010, Takahashi *et al*., 2012, Fraas *et al*., 2014 and Spartz *et al*., 2014; but unlike the published work, we describe the procedures that enable measuring a larger spectrum of processes occurring during growth of hypocotyls; from the macroscopically visible organ elongation, cell wall pH monitored by confocal microscopy to the real-time nuclear auxin signaling visualized by luciferase bioluminescence.

## Materials and Reagents

1. Aluminum foil
2. Razor blades (Gillette Wilkinson™ Sword)
3. Cellophane foil 80 mm diameter (AA Packaging, catalog number: 325 P cellulose film)
4. Black filter paper 90 mm diameter (MACHEREY-NAGEL, catalog number: 409009)
5. Falcon 60 x 15 mm dishes (Corning, catalog number: 353004)
6. 12-well tissue culture plates (TPP Techno Plastic Products, catalog number: 92412)
7. 2-well Lab-Tek™ chambered #1.0 borosilicate cover glass (Thermo Fisher Scientific, catalog number: 155380)
8. *Arabidopsis thaliana* seeds: Col-0, apo-pHusion apoplastic pH marker line (Gjetting *et al*., 2012), auxin responsive promoter driving the expression of the firefly luciferase enzyme marker DR5::LUC (Moreno-Risueno *et al*., 2010)
9. Household bleach (sodium hypochlorite 4.7%)
10. 37% hydrochloric acid (Sigma-Aldrich, catalog number: 435570)
11. MES (Duchefa Biochemie, catalog number: M1503.0100)
12. Sucrose (Sigma-Aldrich, catalog number: 84097-1KG)
13. Potassium hydroxide (KOH) (Merck, catalog number: 105021)
14. Agar, plant cell culture tested (Alfa Aesar, catalog number: H26724)
15. Potassium chloride (KCl) (Sigma-Aldrich, catalog number: P9541-500G)
16. Phytagel (Sigma-Aldrich, catalog number: P8169)
17. 10 mM 3-Indoleacetic acid (IAA) (Sigma-Aldrich, catalog number: I2886-5G) dissolved in ethanol
18. 1 mM D-luciferin (Duchefa Biochemie, catalog number: L1349.0100) dissolved in 1x PBS
19. Chlorine gas (see Recipe 1)
20. Half-strength MS agar media (AM+) (see Recipe 2)
21. Depletion medium (DM) (see Recipe 3)

## Equipment

1. Forceps Dumont #5
2. Binocular dissecting microscope Leica EZ4 (Leica Microsystems, model: Leica EZ4)
3. Flatbed scanner Epson Perfection V370 Photo (Epson, model: V370 Photo)
4. For the bioluminescence dark box:
  Lumazone Manual Stage Dark Box (Photometric, model: LMZ-DRK-BOX)
  Evolve EMCCD camera (Photometric, model: Evolve^®^ 512, catalog number: EVO-512-M-FW-16-AC-RP)
  17 mm fixed lens/0.95 (Edmund Optics, model: 59-832)
  125 mm lens (Thorlabs, model: LA1384-A)
5. Zeiss 700 LSM confocal microscope (ZEISS, model: LSM 700) with a 20x/0.8 Plan-Apochromat M27 objective

## Software

1. Microsoft Excel program (https://products.office.com/en/excel)
2. Fiji program (http://fiji.sc/)
3. MATLAB program (https://www.mathworks.com/products/matlab.html)
4. AutoIt program (https://www.autoitscript.com/site/autoit/)

## Procedure

**Figure 1:**
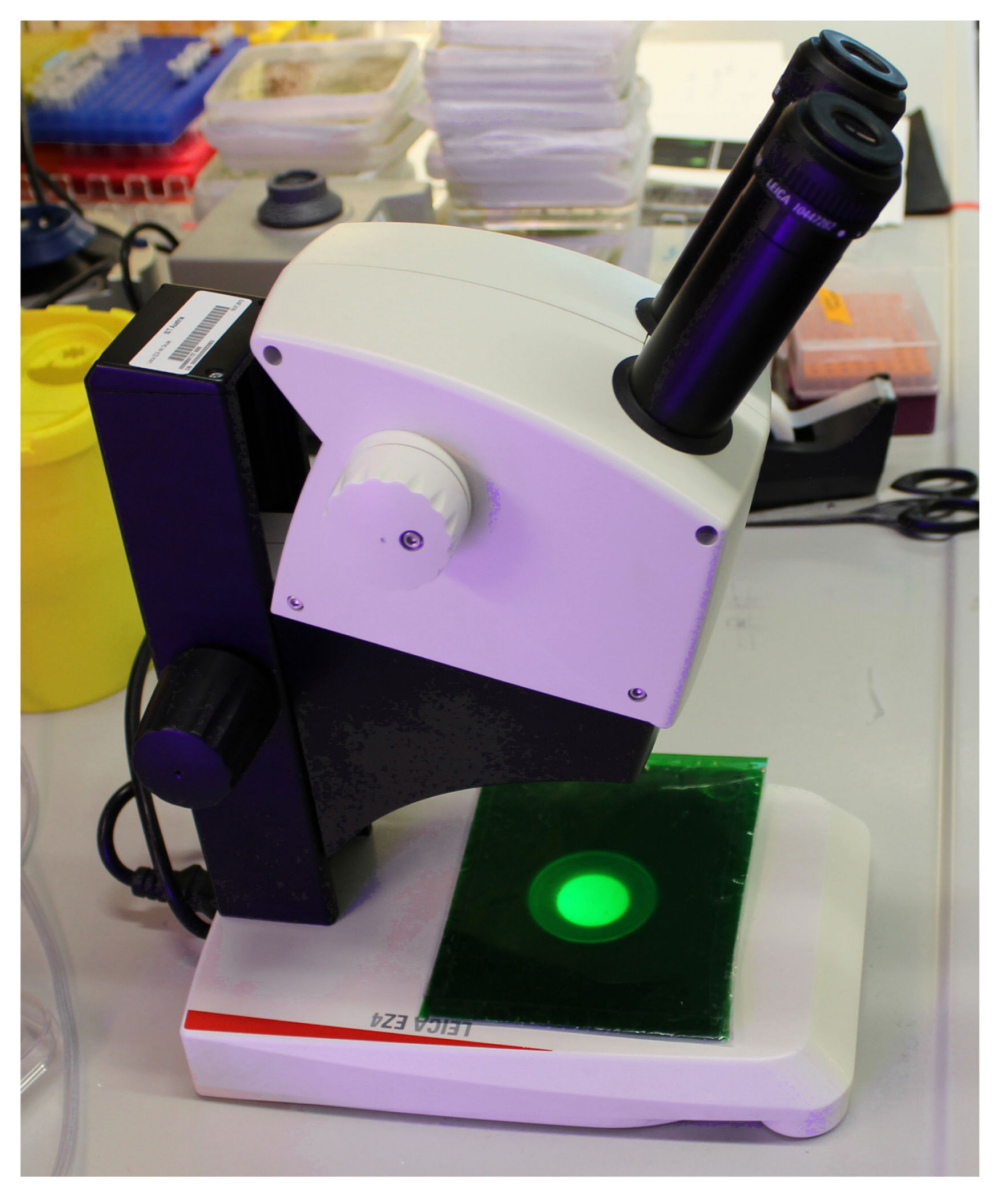
Binocular dissecting microscope with a green filter

**Figure 2:**
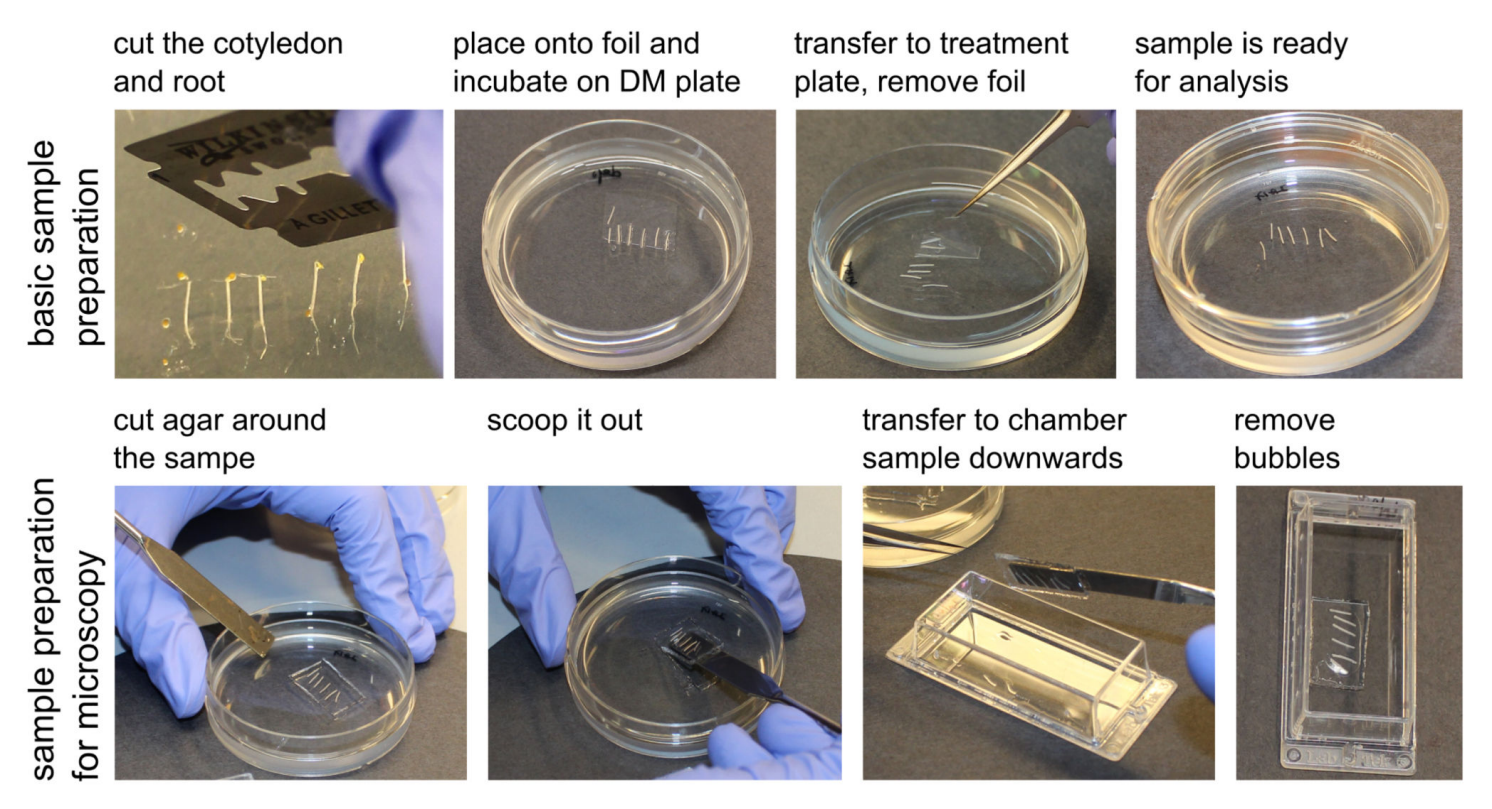
The sample preparation procedure

**Figure 3:**
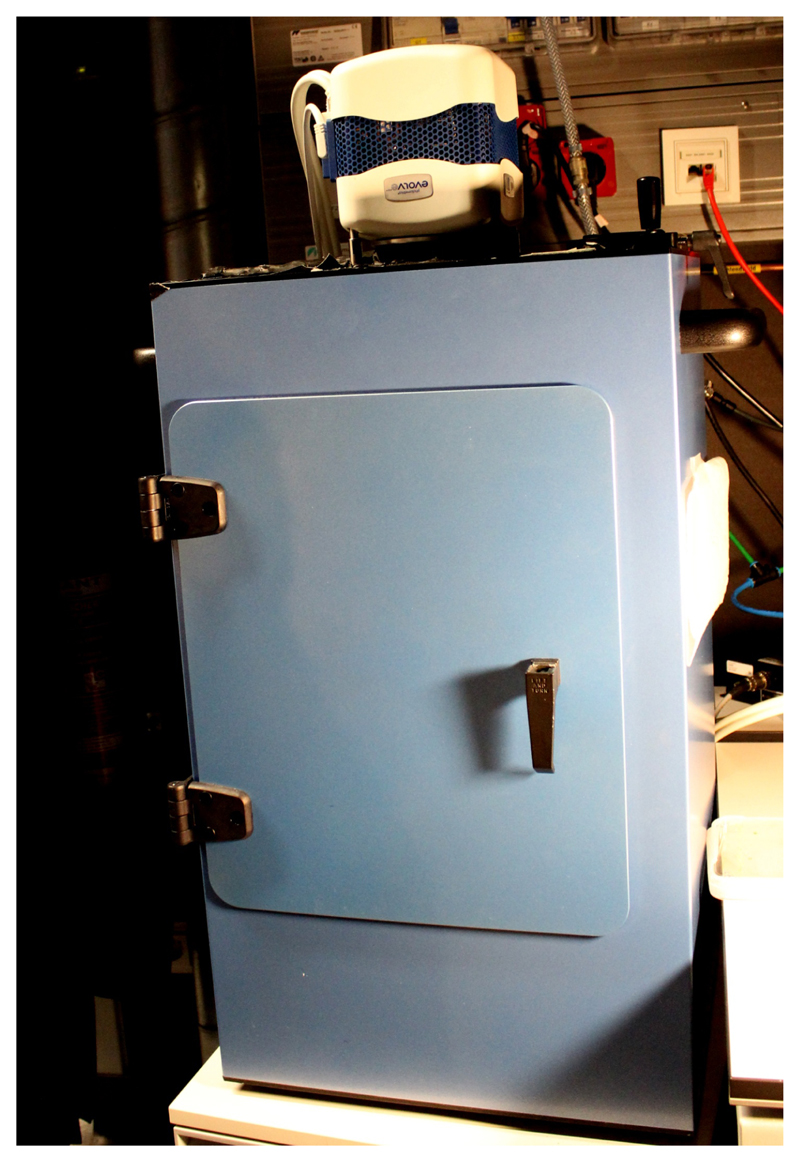
Dark box with a Photometric Evolve EMCCD camera

- Hypocotyl elongation measurement
  1. Surface sterilize Col-0 seeds (or any other genotype of your interest) by chlorine gas (Recipe 1) overnight. Plate the sterilized seeds on the AM+ medium (Recipe 2). Stratify for two days at 4 °C in the dark, then place vertically under light to cultivate for around 6 h in a growth room at 21 °C. Wrap with aluminum foil and grow for another 66 h vertically at 21 °C.
  2. Prepare a depletion plate with 5 ml depletion medium (DM, Recipe 3) in a Falcon 60 x 15 mm dish. After solidification, place cellophane foil onto the surface. Damp the cellophane foil with liquid depletion medium solution.
  3. Place a dissecting microscope in a dark room and cover the illumination with a green filter made of 8 layers of green office foil (Figure 1).
  4. Uncover the Petri dishes with seedlings and select the seedlings with similar hypocotyl length excluding the longest and shortest ones. Decapitate the seedlings right below the apical hook and before the shoot-root junction to get a hypocotyl segment by cutting them on the surface of the agar using a very sharp razor blade. Prepare 6-8 segments for each treatment. Using sharp forceps, transfer the segments onto the cellophane foil in the depletion plate without squeezing them, the sample preparation procedure is depicted in Figure 2. Keep in darkness for 30-60 min.
  5. Afterwards, transfer the segments by flipping the cellophane foil onto a treatment plate with the depletion medium supplemented with the desired treatment (Figure 2), in our case 10 µM 3-Indoleacetic acid (IAA) and the mock control (Ethanol equivalent).
  6. Immediately place the treatment plates on a flatbed scanner, imaging through the layer of the phytagel. A wet black filter paper is placed into the lid of the dish to improve the contrast of the image. Scan the samples in the 8-bit grayscale and at 2,400 dpi every 10 min automatically using the AutoIt program (see Supplemental file 1).
- Measuring the TIR1/AFB-Aux/IAA dependent response using the DR5::LUC marker line
  1. Prepare the 6-10 decapitalized segments of DR5::LUC marker line for each treatment, as described before, on depletion medium. Add around 50 µl of 1 mM D-luciferin dissolved in 1x PBS and immerse the segments entirely for 30 min.
  2. Prepare the treatment solution (DM with desired drugs)–in our case, DM + mock or DM + 10 µM IAA. Pour 3 ml medium into each well of a 12 wells tissue culture plate and let the medium solidify; four wells can be imaged simultaneously.
    *Note: There is no D-luciferin in the treatment solution, the substrate originates from Step B1. This is sufficient for approx. 5-h imaging. Alternatively, one can put D-luciferin in the treatment solution for a longer time of imaging.*
  3. Using the cellophane foil, transfer the segments onto the surface of the treatment medium and remove the foil.
  4. Immediately image in a dark box (Figure 3) with a Photometric Evolve^®^ EMCCD camera equipped with a 17 mm fixed lens/0.95 and an additional 125 mm lens. Set the multiplier EMCCD gain to 150 and the exposure time to 110 sec, and image every 2 min.
- Imaging the apoplastic pH using apo-pHusion apoplastic pH marker line
  1. Prepare the decapitated hypocotyl of the apo-pHusion apoplastic pH marker line as above.
  2. Prepare 5 ml of DM medium with or without 10 µM IAA.
  3. Transfer 5 hypocotyl segments onto the surface of the agar with the treatment. Cut out a piece of the agar with the segments using a spatula. Place the agar with the segments into the Lab-Tek™ chambered cover glass so that the segments are placed between the cover glass and the agar. When using the 2-well chambered glass, a treatment and a control sample can be imaged simultaneously.
  4. Alternatively, the treatment can be very carefully pipetted to the hypocotyl segments during imaging. Then ~50 μl DM with the treatment can be used, but one must be extremely careful not to move the sample during imaging.
  5. Using a confocal microscope with a 20x/0.8 Plan-Apochromat M27 objective, set the position of each segment using the position manager so that the apical region of the hypocotyl segment is imaged. Image 5 z-sections, z-thickness matched to the pinhole size, of each hypocotyl segment.
  6. Set the microscope for simultaneous imaging of GFP and RFP by exciting using 488 and 555 nm diode lasers, and splitting the emitted light with a short pass 550 nm and long pass 560 nm filters, 16 bits per pixel. Image all positions every 5 min.

## Data analysis

**Figure 4:**
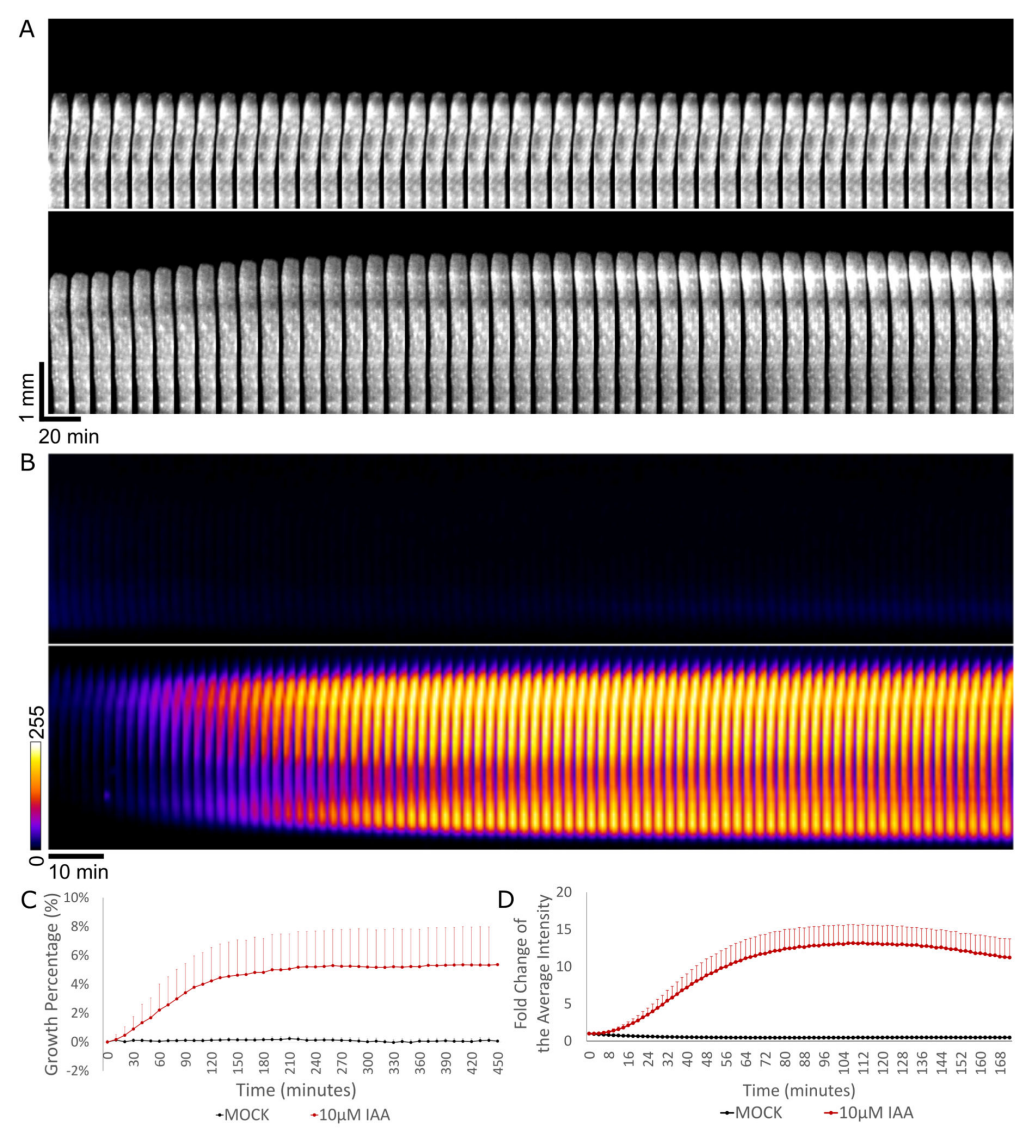
Hypocotyl segment growth and DR5::LUC intensity measurements. A. The kymograph of the hypocotyl segment of Col-0 under mock treatment (upper row) and 10 μM IAA treatment (lower row) from 0 to 460 min; time interval of 10 min. The kymograph was done by making montage of the growing part of one representative sample in Fiji. (Note that to visualize the growth better, the upper half part of the hypocotyl where growth takes place was used for making montage). Vertical and horizontal scale bars represent 1 mm and 20 min, respectively. B. The kymograph of the luminescence intensity in the mock-treated DR5::LUC hypocotyls (upper row) and 10 μM IAA-treated (lower row) from 0 to 172 min; time interval of 2 min. The ‘FIRE’ look-up table was applied in Fiji. Scale bar is 10 min. C. Quantification of the growth of Col-0 hypocotyls treated with mock or 10 μM IAA from 0 to 460 min. The growth is expressed as the percentage of the original segment length. D. Quantification of the luminescence intensity in the DR5::LUC hypocotyls treated with mock or 10 μM IAA. The Fold change is the average intensity of the hypocotyls normalized by the intensity at timepoint 0.

**Figure 5:**
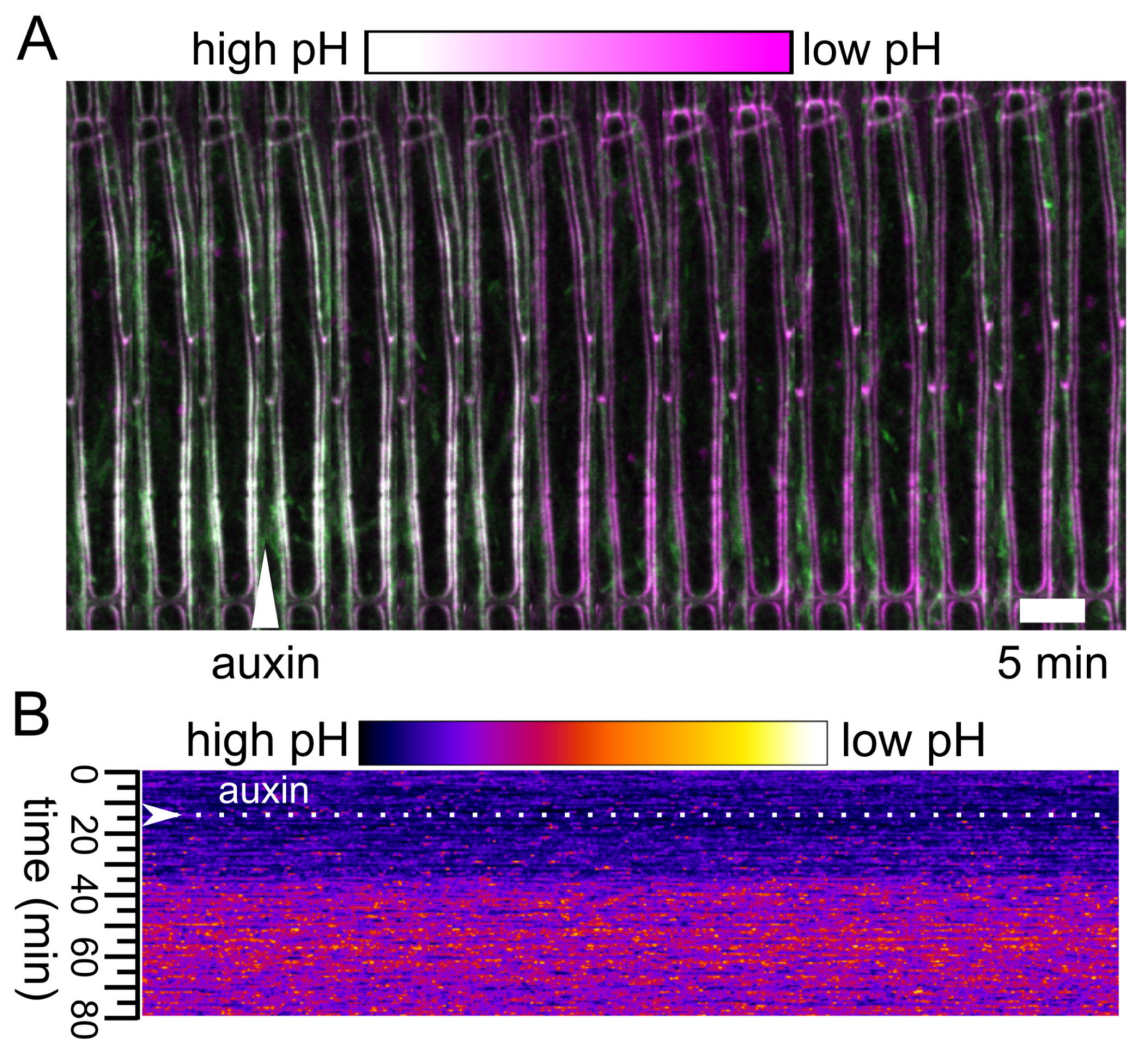
Analysis of the apoplast pH. A. The apoplast pH after application of auxin to a hypocotyl expressing the apo-pHusion sensor. Auxin application is indicated with an arrowhead, GFP is shown in green while RFP in magenta. B. The output of the AreaKymo MATLAB^®^ script. Time is progressing from top to bottom; ratio of the two fluorophores is shown using the ‘FIRE’ look-up-table.

- Hypocotyl elongation image analysis
  1. To achieve unbiased measurement, we created a Fiji macro (see Supplemental file 1) for analyzing the length of the segment at each time point. The macro firstly creates the time lapse of the image sequence captured from the scanner, then allows you to manually create a rectangle ROI for each segment, followed by automatically thresholding each ROI and measuring the Feret’s diameter, the maximum caliper, as the length of the segment. The macro eventually generates ‘.txt’ file for each ROI or hypocotyl, including the Feret’s diameter of that hypocotyl in each time point.
  2. Copy and paste the result into Excel, set the initial length of the segment as 100%, and calculate the length of the hypocotyl at each time point to obtain a growth curve (Figure 4C). Besides, growth can be visualized by creating a montage-kymograph of individual hypocotyl segment in Fiji (Figure 4A).
- Analysis of the bioluminescence intensity
  1. Analyze the image sequence in Fiji (Schindelin *et al*., 2012). Manually outline all the segments via Polygon selection, at their brightest time frame, and add them into the region of interest (ROI) manager, followed by multi-measuring the mean grey value. This gives the average intensity of each segment at each time point.
  2. Copy and paste the result into Excel. Take the initial intensity of the segment as 100%, and analyze the average of the luminescence intensity of each hypocotyl at each time point, to get an intensity curve in time. Additionally, one can visualize the growth and the luminescence intensity by creating a montage-kymograph in Fiji (Figures 4B and 4D).
- Image analysis of the cell wall pH
  1. Analyze the apoplastic pH using Fiji. We use the SUM projections of the z-stacks (Figure 5A). Set the threshold of the apoplast region using the RFP channel so that only the cell wall signal is selected. Create the selection using the ‘create selection’ command and measure intensity in GFP and RFP channels. Analyze the intensity ratios in Excel program. We analyze the apoplastic pH change in relative values the lower the GFP intensity, the lower the apoplastic pH is (Gjetting *et al*., 2012).
  2. Alternatively to Step C1, the apoplastic pH can be visualized and measured using the AreaKymo MATLAB^®^ script, Figure 5B (Supplemental file 1). The AreaKymo script essentially does the same procedure as described in Step C1, but does so automatically without the user input, allowing for rapid processing of several hypocotyls at a time. The user first merges several hypocotyl SUM projection time series into one ‘tif.’ series using the Fiji program (‘combine stacks’ command) and converts them into 16-bit ‘tif.’ images (MATLAB does not handle well the 32-bit images that the SUM projection command creates). Then the user should find a threshold of the RFP channel that is optimal for selecting just the apoplast signal. In MATLAB, the AreaKymo script is run, the combined time series is selected, and the user specifies the value of the threshold for the RFP channel and the desired width of the rectangle that will represent the individual timeframe. The script outputs the visual representation of apoplastic pH and also the values in the form of a series of boxplots.

## Notes

During all steps where the hypocotyls are manipulated (cut and transferred to new plates) it is crucial to be extremely gentle with the tissue, not squeeze it but rather scoop it using sharp forceps. The tissue needs to be protected from drying; plates must be kept closed whenever possible to prevent excessive evaporation.

## Recipes

1. Chlorine gas sterilization
  100 ml household bleach
  4.5 ml 37% HCl
2. Half-strength MS agar media (AM+)
  Half Murashige and Skoog Basal Salts
  1% sucrose
  Adjust pH to 5.8 by KOH
  0.8% agar, plant cell tested
  MiliQ water as solvent
3. Depletion medium (DM)
  10 mM KCl
  1 mM MES
  Adjust pH to 6 by KOH
  1.5% phytagel
  MiliQ water as solvent
  *Note: The phytagel brings better transparency than normal agar, contributing to the better quality of the scanning.*
